# Involvement of catalase and superoxide dismutase in hydrophobic organic solvent tolerance of *Escherichia coli*

**DOI:** 10.1186/s13568-021-01258-w

**Published:** 2021-06-29

**Authors:** Noriyuki Doukyu, Katsuya Taguchi

**Affiliations:** 1 Department of Life Science, Toyo University, 1-1-1 Izumino, Itakura-machi, Gunma 374-0193 Japan; 2 Bio-Nano Electronic Research Center, Toyo University, 2100, Kujirai, Kawagoe, Saitama 350-8585 Japan

**Keywords:** *Escherichia coli*, Organic solvent tolerance, Catalase, Superoxide dismutase, Reactive oxygen species

## Abstract

*Escherichia coli* strains are generally sensitive to hydrophobic organic solvents such as *n*-hexane and cyclohexane. Oxidative stress in *E*. *coli* by exposure to these hydrophobic organic solvents has been poorly understood. In the present study, we examined organic solvent tolerance and oxygen radical generation in *E. coli* mutants deficient in reactive oxygen species (ROS)-scavenging enzymes. The organic solvent tolerances in single gene mutants lacking genes encoding superoxide dismutase (*sodA*, *sodB*, and *sodC*), catalase (*katE* and *katG*), and alkyl hydroperoxide reductase (*ahpCF*) were similar to that of parent strain BW25113. We constructed a BW25113-based *katE katG* double mutant (BW25113∆*katE*∆*katG*) and *sodA sodB* double mutant (BW25113*sodA*∆*sodB*). These double-gene mutants were more sensitive to hydrophobic organic solvents than BW25113. In addition, the intracellular ROS levels in *E*. *coli* strains increased by the addition of *n*-hexane or cyclohexane. The ROS levels in BW25113∆*katE*∆*katG* and BW25113∆*sodA*∆*sodB* induced by exposure to the solvents were higher than that in BW25113. These results suggested that ROS-scavenging enzymes contribute to the maintenance of organic solvent tolerance in *E. coli*. In addition, the promoter activities of *sodA* and *sodB* were significantly increased by exposure to *n*-hexane.

**Supplementary Information:**

The online version contains supplementary material available at 10.1186/s13568-021-01258-w.

## Introduction

Efficient microbial production of valuable organic compounds including biofuels and fine chemicals from renewable biomass resources is one of the crucial challenges in the establishment of a sustainable society. Some of these valuable chemicals such as advanced biofuels and bulk chemicals, including organic solvents including 1-octanol and styrene, are toxic to various microorganisms such as *E*. *coli* and *Pseudomonas putida* (Akhtar et al. 2015; Lennen et al. 2013; Mukhopadhyay 2015). Accumulation of these toxic products can negatively impact the viability of microbes and impede their efficient mass production of organic compounds (Doukyu and Iida 2020; Doukyu et al. 2003; Mukhopadhyay 2015). Bacterial growth and stress response have been studied in a variety of organic solvents (Heipieper et al. 2007; Inoue and Horikoshi 1989; Ramos et al. 2002). Solvents with higher hydrophobicity tend to increase in toxicity (Kabelitz et al. 2003). At saturated concentrations, the toxicity of the hydrophobic organic solvent is inversely correlated with the log*P*_ow_ of the solvent (common logarithm of partition coefficient of given solvent in a mixture of n-octanol and water) (Inoue and Horikoshi 1989). In the log*P*_ow_ range of 2 to 4, increasing the hydrophobicity of the solvent could enhance the level of toxicity (Kabelitz et al. 2003). The effects of organic solvents on membrane structure and fluidity differ depending on the polarity of the solvent (Griepernau et al. 2007).

It has been reported that reactive oxygen species (ROS) are generated in *Escherichia coli* during exposure to hydrophilic solvents such as ethanol and *n*-butanol (Cao et al. 2017; Chin et al. 2013; Rutherford et al. 2010). A ROS assay using a cell-permeant fluorescent dye showed a significant increase in intracellular ROS levels in *n*-butanol-exposed *E*. *coli* cells (Rutherford et al. 2010). In addition, an *E*. *coli* strain expressing metallothioneins, which function in ROS scavenging, exhibited an increased growth rate under *n*-butanol stress (Chin et al. 2013). Thus, ROS generation has been suggested to be attributable in part to the toxicity of hydrophilic organic solvents. On the other hand, various tolerance mechanisms in *E*. *coli* strains have been studied with hydrophobic organic solvents such as *n*-hexane and cyclohexane (Aono 1998). These findings are useful for improving the production of hydrophobic solvents and fatty acids (Akhtar et al. 2015; Lennen et al. 2013). However, little is known about the ROS generation in microbial cells by these hydrophobic organic solvents.

Various ROS, including superoxide, H_2_O_2_ and hydroxyl radical, are generated as by-products in cells grown aerobically. Specific enzymes such as catalase and superoxide dismutase (SOD) decrease the levels of cytotoxic ROS. *E*. *coli* harbors two different catalase genes: *katG*, which encodes hydroperoxidase I (HPI) (Triggs-Raine et al. 1988) and *katE*, which encodes HPII (Mulvey et al. 1988). HPI is induced by H_2_O_2_ in an OxyR-dependent manner, while expression of HPII is dependent on a sigma factor, RpoS (Mukhopadhyay and Schellhorn 1994). The activities of both catalases increase as the growing cells enter stationary phase. In addition, the genome of *E*. *coli* includes the alkyl hydroperoxide reductase gene (*ahpCF*), which scavenges H_2_O_2_ and organic hydroperoxides (Ferrante et al. 1995; Smillie et al. 1992). *E*. *coli* possesses three distinct SOD genes: *sodA*, which encodes a cytosolic manganese-containing SOD (Touati 1983), *sodB*, which encodes a cytosolic iron-containing SOD (Sakamoto and Touati 1984) and *sodC*, which encodes a periplasmic copper and zinc-containing SOD (Imlay and Imlay 1996). SodC is induced in stationary phase and seems to be important to protect the cell from exogenous ROS attacks (Gort Amy et al. 2002).

Both the *katE katG* double mutant and *sodA sodB* double mutant are sensitive to H_2_O_2_ due to DNA damage (Imlay and Linn 1987; Ruiz-Laguna and Pueyo 1999). In addition, ROS levels significantly increased in *sodA sodB* double mutant by the addition of *p*-nonylphenol (Okai et al. 2004). Thus, it was suggested that one of the primary actions of *p*-nonylphenol in cells is the generation of superoxide.

In this study, we examined the involvement of catalase and SOD genes in organic solvent-tolerance in *E. coli*. We found that two BW25113-based mutants, the ∆*katE*∆*katG* mutant and ∆*sodA*∆*sodB* mutant, were highly sensitive to organic solvents. In addition, it was shown that the increases in intracellular ROS levels in these two mutants were larger than that in strain BW25113 when these strains were treated with hydrophobic organic solvents. These results showed that catalase and SOD are implicated in the organic solvent tolerance in *E*. *coli*.

## Materials and methods

### Media, culture conditions and materials

*E*. *coli* strains were grown aerobically at 37 °C in LBGMg medium consisting of 1% tryptone, 0.5% yeast extract, 1% NaCl, 0.1% glucose, and 10 mM MgSO_4_ (Aono et al. 1991). The LBGMg medium was solidified with 1.5% (wt/vol) agar. Ampicillin (50 μg/ml) or kanamycin (50 μg/ml) was added to the medium when necessary. Lysogeny broth (LB) agar medium was used for measuring colony-forming units (Neidhardt et al. 1974). M9 medium was used for ROS assay (Neidhardt et al. 1974). Growth of cells in liquid culture was monitored by measuring the optical density at 660 nm (OD_660_). The *tert*-butyl hydroperoxide was obtained from FUJIFILM Wako Pure Chemical Industries (Osaka, Japan). The organic solvents used were of the highest quality available (FUJIFILM Wako Pure Chemical Industries). The log*P*_ow_ values of the hydrophobic solvents used in this study were as follows: cyclooctane (log*P*_ow_, 4.1), *n*-hexane (log*P*_ow_, 3.9) and cyclohexane (log*P*_ow_, 3.4).

### Bacterial strains and plasmids

The *E. coli* strains and plasmids used in this study are summarized in Tables 1 and 2, respectively. Strain BW25113 and its single-gene knockout mutants were obtained from the National Bio-Resource Project (NIG, Mishima, Japan) (Baba et al. 2006). The plasmid pCP20 was also supplied by NIG. pMC1403 contains a sequence downstream of the 10th codon of *lacZ*, but it does not contain the *lacZ* promoter, the Shine-Dalgarno sequence, and the start codon (Casadaban et al. 1980). The plasmid pMW119 was purchased from Nippon Gene (Tokyo).

**Table 1:** *Escheria* *coli* strains used in this study

Strain	JW ID^a^	Relevant characteristics	References
BW25113		*lacI*^q^ *rrnB*_T14_ lacZ_WJ16_ *hsdR*514 *araBAD*_AH33_ *rhaBAD*_LD78_	(Baba et al. 2006)
BW25113∆*katE*	JW1721	Same as BW25113, but with *katE*::Km^R^	(Baba et al. 2006)
BW25113∆*katG*	JW3914	Same as BW25113, but with *katG*::Km^R^	(Baba et al. 2006)
BW25113∆*sodA*	JW3879	Same as BW25113, but with *sodA*::Km^R^	(Baba et al. 2006)
BW25113∆*sodB*	JW1648	Same as BW25113, but with *sodB*::Km^R^	(Baba et al. 2006)
BW25113∆*sodC*	JW1638	Same as BW25113, but with *sodC*::Km^R^	(Baba et al. 2006)
BW25113∆ahpF	JW0599	Same as BW25113, but with *ahpF*::Km^R^	(Baba et al. 2006)
BW25113∆*katE*∆*katG*		Same as BW25113, but with ∆*katE* and ∆*katG*	This study
BW25113∆*sodA*∆*sodB*		Same as BW25113, but with ∆*sodA* and ∆*sodB*	This study
BW25113(pMW119)		BW25113 harboring pMW119	This study
BW25113∆*katE*∆*katG*(pMW119)		BW25113∆*katE*∆*katG* harboring pMW119	This study
BW25113∆*katE*∆*katG*(pMWkatE)		BW25113∆*katE*∆*katG* harboring pMWkatE	This study
BW25113∆*katE*∆*katG*(pMWkatG)		BW25113∆*katE*∆*katG* harboring pMWkatG	This study
BW25113∆*katE*∆*katG*(pMWkatEkatG)		BW25113∆*katE*∆*katG* harboring pMWkatEkatG	This study
BW25113∆*sodA*∆*sodB*(pMW119)		BW25113∆*sodA*∆*sodB* harboring pMW119	This study
BW25113∆*sodA*∆*sodB*(pMWsodA)		BW25113∆*sodA*∆*sodB* harboring pMWsodA	This study
BW25113∆*sodA*∆*sodB*(pMWsodB)		BW25113∆*sodA*∆*sodB* harboring pMWsodB	This study
BW25113∆*sodA*∆*sodB*(pMWsodAsodB)		BW25113∆*sodA*∆*sodB* harboring pMWsodAsodB	This study
BW25113(pMCkatEp)		BW25113 harboring pMCkatEp	This study
BW25113(pMCkatGp)		BW25113 harboring pMCkatGp	This study
BW25113(pMCsodAp)		BW25113 harboring pMCsodAp	This study
BW25113(pMCsodBp)		BW25113 harboring pMCsodBp	This study

^a^JW ID of the Keio Collection by the National Bio-Resource Project (NIG, Mishima, Japan): *E. coli* (Baba et al. 2006)

**Table 2:** Plasmids used in this study

Plasmids	Relevant characteristics	References
pCP20	pSC101-based vector expressing the Flp recombinase with *repA*(Ts), Amp^r^, Cm^r^	(Cherepanov and Wackernagel 1995)
pMW119	Expression vector with the replication origin of pSC101, Amp^r^	Nippon Gene
pMWkatE	pMW119-based plasmid carrying *katE*	This study
pMWkatG	pMW119-based plasmid carrying *katG*	This study
pMWkatEkatG	pMW119-based plasmid carrying *katE* and *katG*	This study
pMWsodA	pMW119-based plasmid carrying *sodA*	This study
pMWsodB	pMW119-based plasmid carrying *sodB*	This study
pMWsodAsodB	pMW119-based plasmid carrying *sodA* and *sodB*	This study
pMC1403	Cloning vector for the *lacZ* reporter system with the replication origin of pMB1, Amp^r^	(Casadaban et al. 1980)
pMCkatEp	pMC1403-based plasmid carrying the *katE* promoter region	This study
pMCkatGp	pMC1403-based plasmid carrying the *katG* promoter region	This study
pMCsodAp	pMC1403-based plasmid carrying the *sodA* promoter region	This study
pMCsodBp	pMC1403-based plasmid carrying the *soxB* promoter region	This study

### Construction of BW25113∆*katE*∆*katG* and BW25113∆*sodA*∆*sodB*

The Km^R^ cassettes in BW25113∆*sodB* and BW25113∆*katE* were eliminated with pCP20 (Cherepanov and Wackernagel 1995). Elimination of the Km^R^ cassette was confirmed by PCR analysis using chromosomal DNA. The combination of primers for BW25113∆*sodB* was sodB-S and sodB-AS, and that for BW25113∆*katE* was katE-S and katE-AS (Table 3). BW25113∆*sodA*∆*sodB* and BW25113∆*katE*∆*katG* were constructed from the Km^R^ cassette-eliminated mutants BW25113∆*sodB* and BW25113∆*katE* by P1 transduction of kanamycin-resistance with BW25113∆*sodA* and BW25113∆*katG* as the donor, respectively. The Km^R^ cassettes in BW25113∆*sodA*∆*sodB* and BW25113∆*katE*∆*katG* were also eliminated with pCP20. Elimination of the Km^R^ cassette in the *sodA* and *katG* region was confirmed by PCR analysis. The combination of primers for the *sodA* disruption was sodA-S and sodA-AS, and that for the ∆*katG* disruption was katG-S and katG-AS.

**Table 3:** Primers used in this study

Primer	Sequence (5′ to 3′)	Positions
katE-S	TACTCAGTCACTTCCCCTTC	426–445 bp upstream of the initiation codon of *katE*
katE-AS	AACTACGGCATTATCGAGGC	934–953 bp downstream of the stop codon of *katE*
katG-S	GGGGCAGATTAACGTTTCGT	1141–1160 bp upstream of the initiation codon of *katG*
katG-AS	GCCAGCACAATCAGCACAAT	924–943 bp downstream of the stop codon of *katG*
sodA-S	CGATGTTAGCGGCGACAATA	1277–1296 bp upstream of the initiation codon of *sodA*
sodA-AS	GCTCTGGCTTTGACTTTACG	1190–1209 bp downstream of the stop codon of *sodA*
sodB-S	TTCGATCACGCTCTGTGCTT	904–923 bp upstream of the initiation codon of *sodB*
sodB-AS	TTAACTATCCGTTGCTGGCG	1305–1324 bp downstream of the stop codon of *sodB*
katEc-S	AAAGTCGACATTTGCCACGCAGCATCCAG	311–330 bp upstream of the initiation codon of *katE*, SalI site underlined
katEc-AS	TTTGGTACCAGGCCGGATAAGGCGTTCAC	69–88 bp downstream of the stop codon of *katE*, KpnI site underlined
katGc-S	AAAGGTACCTTACGCGATTTGCCATACGC	352–371 bp upstream of the initiation codon of *katG*, KpnI site underlined
katGc-AS	TTTGAGCTCGTGTGTAGTTTTCGTTCGCC	63–82 bp downstream of the stop codon of *katG*, SacI site underlined
sodAc-S	AAAGCATGCTAAAAACAGGCTGCACTGGC	333–352 bp upstream of the initiation codon of *sodA*, SphI site underlined
sodAc-AS	TTTGTCGACTTTTTTAAGCTGATATGCGGCC	32–53 bp downstream of the stop codon of *sodA*, SalI site underlined
sodBc-S	AAAGTCGACCTCTCAGTGAAGACTACTGG	182–201 bp upstream of the initiation codon of *sodB*, SalI site underlined
sodBc-AS	TTTGGATCCTGCCTTATCCGACCTACATC	63–82 bp downstream of the stop codon of *sodB*, BamHI site underlined
katEp-S	AAAGAATTCAACCGGGAGGTATGTAATCC	446–466 bp upstream of the initiation codon of *katE*, EcoRI site underlined
katEp-AS	TTTGGATCCTGCTGATGTGGGTTCTTTTCG	15–35 bp downstream of the initiation codon of *katE*, BamHI site underlined
katGp-S	AAACCCGGGCGAATATTGCCATGGATATGG	439–459 bp upstream of the initiation codon of *katG*, SmaI site underlined
katGp-AS	TTTGGATCCGTGGTGTTATGGATATCGTCTG	11–32 bp downstream of the initiation codon of *katG*, BamHI site underlined
sodAp-S	AAAGAATTCGCCCAGAAATTCGGTAGTAAC	451–472 bp upstream of the initiation codon of *sodA*, EcoRI site underlined
sodAp-AS	TTTGGATCCTCCAGGGCATCGTAAGCATACG	26–47 bp downstream of the initiation codon of *sodA*, BamHI site underlined
sodBp-S	AAAGAATTCGTACCGGTTTTGATTGCAGC	434–453 bp upstream of the initiation codon of *sodB*, EcoRI site underlined
sodBp-AS	TTTGGATCCGCCAGAGCATCTTTAGCATAT	27–47 bp downstream of the initiation codon of *sodA*, BamHI site underlined

### Measurement of organic solvent-tolerance in *E. coli*

Cultures of *E. coli* strains in LBGMg medium after 16 h of incubation (OD_660_, 4 to 5) at 30 °C were diluted with 0.8% saline by serial tenfold dilutions. Five microliters of each suspension was spotted on LBGMg agar medium. The agar surface was overlaid with organic solvents (Tsukagoshi and Aono 2000). The formation of colonies on the agar was observed after 48 h of incubation at 25 °C.

### Cloning of the *katE*, *katG*, *sodA* and *sodB* genes

The regions of *katE*, *katG*, *sodA* and *sodB* were amplified by PCR using AccuPrime Taq DNA Polymerase (Thermo Fisher Scientific Inc.) with high fidelity and BW25113 chromosomal DNA as the template. The primers used were designed according to the genome sequence of BW25113 deposited in GenBank (accession number CP009273). The combination of primers for *katE* was katEc-S and katEc-AS, that for *katG* was katGc-S and katGc-AS, that for *sodA* was sodAc-S and sodAc-AS, and that for *sodB* was sodBc-S and sodBc-AS (Table 3). A restriction endonuclease cleavage site was introduced into all primer sequences. The amplified fragments were digested with the relevant restriction enzymes and ligated into the cloning site of pMW119 under the same direction as the *lac* promoter to construct plasmids pMWkatE, pMWkatG, pMWsodA, and pMWsodB, respectively. The pMWkatE was digested with KpnI and SacI, and then the fragments containing *katE* were ligated between KpnI and SacI sites of pMWkatG. The resulting plasmid was designated pMWkatEkatG. In addition, the pMWsodA was digested with SphI and SalI, and then the fragments containing *sodA* were ligated between SphI and SalI sites of pMWsodB. The resulting plasmid was designated pMWsodAsodB.

### Enzyme activity assay

*E*. *coli* cells grown in LBGMg medium after incubation at 30 °C for 16 h were harvested by centrifugation (4400×*g* for 10 min at 4 °C) and suspended in 10 mM of Tris–HCl buffer (pH 8.0). The cell suspension was sonicated on ice and centrifuged (10,000×*g*, 10 min at 4 °C). The supernatant was used for the enzyme activity assay.

Catalase activity was determined by following the rate of H_2_O_2_ consumption at 240 nm (Claiborne et al. 1979). The enzyme activity was calculated from the molar adsorption coefficient of the H_2_O_2_ (ε = 43.6 M^−1^ cm^−1^). The reaction mixture (1 mL) contained 50 mM potassium phosphate buffer at pH 7.0 and 10 mM H_2_O_2_. The reaction was initiated by adding the enzyme solution (50 μL) to the reaction mixture and the initial velocity of H_2_O_2_ disappearance was measured at 30 °C. One unit of enzyme activity was defined as the amount of enzyme that decomposes 1 μmol of H_2_O_2_ per min.

SOD activity was measured by following the rate of pyrogallol autooxidation (Marklund and Marklund 1974). The enzyme solution (10 μL) was mixed with 2.48 mL of 50 mM Tris–HCl and 1 mM EDTA at pH 8.2. After preincubation for 5 min at 25 °C, the reaction was started by adding 10 μL of 50 mM pyrogallol solution in 10 mM HCl. The change of absorbance was monitored at 325 nm. One unit of enzyme is defined as the amount of enzyme that inhibits the autoxidation rate of pyrogallol by 50%.

### Protein content

The protein concentration was determined by the method of Bradford (Bradford 1976) using bovine serum albumin as the standard.

### Sensitivities of *E*. *coli* strains to H_2_O_2_ and menadione

*E. coli* cells grown in LBGMg medium (10 mL) at 37 °C to an OD_660_ of about 0.6 (approximately 4 to 5 × 10^8^ cells/ml) were harvested by centrifugation (4400×g for 10 min at 4 °C), and suspended in PBS buffer (10 mL) consisting of 140 mM NaCl, 8.1 mM Na_2_HPO_4_, 2.7 mM KCl, and 1.5 mM KH_2_PO_4_ (pH 7.4). The cell suspension was washed once by centrifugation, resuspended in PBS buffer with H_2_O_2_ (1 to 4 mM) or menadione (15 to 60 mM), and further incubated with shaking at 37 °C. After incubation for 1 h, each suspension was plated on LB agar medium. The number of colonies formed on the agar plate was counted after 24 h of incubation at 30 °C.

### Detection of reactive oxygen species

The ROS were detected with the 5-(and-6)-carboxy-2’,7’-dichlorodihydrofluorescein diacetate (carboxy-H_2_DCFDA) (Molecular Probes, Eugene, OR) as reported previously with a slight modification (Rutherford et al. 2010). Carboxy-H_2_DCFDA is a cell-permeable indicator for ROS that does not fluoresce until it is hydrolyzed by esterases and oxidation occurs within cells. A 100 μl culture of overnight-grown *E*. *coli* cells was inoculated onto 10 ml of fresh LBGMg medium and incubated at 37 °C with shaking. After incubation for 3 h, 0.5 mL of 7.78 M *tert*-butyl hydroperoxide (TBHP; used as a positive control for oxidative stress) or 1 mL of an organic solvent (*n*-hexane or cyclohexane) was added to the culture. The culture was further incubated at 37 °C for 3 h with shaking. Twenty microliters of each culture was added to 1 ml of M9 medium. After incubation at 37 °C for 45 min, 50 μl of 25 mM carboxy-H_2_DCFDA samples was added to the medium. After incubation at 37 °C for 10 min, the OD_660_ and the fluorescence excitation/emission at 485/535 nm of each sample were measured by spectrofluorometer (RF-6000; Shimadzu Co., Kyoto, Japan). Specific fluorescence was calculated as fluorescence/OD_660_.

### Construction of the *lacZ* reporter fusions

The promoter regions of *katE*, *katG*, *sodA* and *sodB* were amplified by PCR using AccuPrime Taq DNA Polymerase and BW25113 chromosomal DNA as the template. The combination of primers for the *katE* promoter region was katEp-S and katEp-AS, that for the *katG* promoter region was katGp-S and katGp-AS, that for the *sodA* promoter region was sodAp-S and sodAp-AS, and that for the *sodB* promoter region was sodBp-S and sodBp-AS (Table 3). The amplified fragments were digested with the relevant restriction enzymes and ligated into the cloning site of pMC1403 to construct plasmids pMCkatEp, pMCkatGp, pMCsodAp, and pMCsodBp, respectively.

### Assay for plasmid-borne β-galactosidase activity

*E*. *coli* strains were grown in LBGMg containing 50 μg/ml ampicillin at 37 °C. Cells in the exponential phase of growth were treated with a small volume of chloroform and assayed for β-galactosidase activity as described previously (Miller 1972).

## Results

### Organic solvent-tolerances of *E. coli* mutants deficient in ROS-scavenging enzymes

**Fig. 1:**
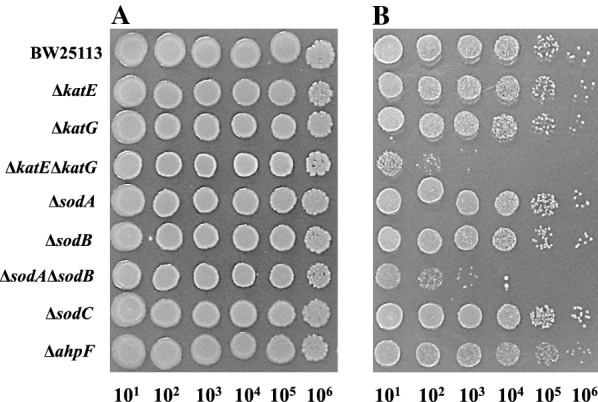
Colony-forming efficiency of *E. coli* BW25113 and its mutants deficient in ROS-scavenging enzymes on LBGMg agar medium. Each strain was grown in the absence of an organic solvent (**A**) and in the presence of *n*-hexane (**B**). Each strain was spotted at a tenfold dilution and incubated at 25 °C for 48 h

The colony-forming efficiency of the BW25113-based ∆*katE*, ∆*katG*, ∆*ahpF*, ∆*sodA*, ∆*sodB*, ∆*sodC*, ∆*katE*∆*katG* and ∆*sodA*∆*sodB* mutants was investigated using an LBGMg agar plate in the presence of *n*-hexane (Fig. 1). All strains formed colonies in all spots on the plate without any solvent. The colony-forming efficiencies in the single gene mutants were similar to that in the parent strain BW25113 in the presence of *n*-hexane. In contrast, the double gene mutants, BW25113∆*katE*∆*katG* and BW25113∆*sodA*∆*sodB*, were highly sensitive to *n*-hexane, exhibiting 10^2^- or 10^3^-fold lower colony-forming efficiencies than the parent strain in the presence of the solvent. These results indicated that catalase and SOD were involved in the maintenance of organic solvent-tolerance in *E*. *coli*.

**Fig. 2:**
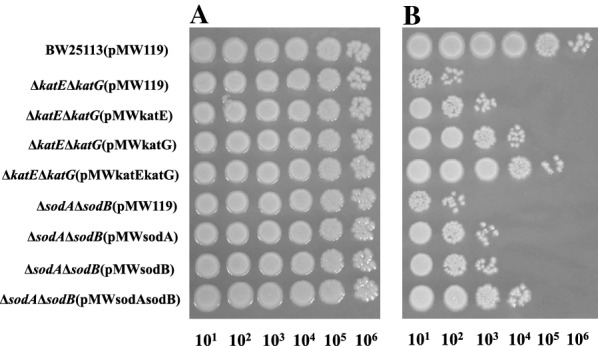
Colony-forming efficiency of BW25113-based recombinant *E. coli* strains. Each strain was grown on LBGMg agar medium containing ampicillin (50 μg/ml) and isopropyl-β-d-thiogalactopyranoside (IPTG; 0.5 mM) in the absence of an organic solvent (**A**) and in the presence of *n*-hexane (**B**). Each strain was spotted at a tenfold dilution and incubated at 25 °C for 48 h

Complementation of organic solvent-tolerances of BW25113∆*katE*∆*katG* and BW25113∆*sodA*∆*sodB* by transformation of the catalase- or SOD-coding gene was investigated (Fig. 2). The colony-forming efficiencies in BW25113∆*katE*∆*katG*(pMWkatE), BW25113∆*katE*∆*katG*(pMWkatG), and BW25113∆*katE*∆*katG*(pMWkatEkatG) were about 10-, 10^2^-, and 10^3^-fold higher than that in BW25113(pMW119) in the presence of *n*-hexane, respectively. On the other hand, the colony-forming efficiencies in BW25113∆*sodA*∆*sodB*(pMWsodA) and BW25113∆*sodA*∆*sodB*(pMWsodB) were both about tenfold higher than that in BW25113(pMW119). In addition, the efficiency in BW25113∆*sodA*∆*sodB*(pMWsodAsodB) was about 10^3^-fold higher than that in BW25113(pMW119). Thus, it was shown that *katE*, *katG*, *sodA*, and *sodB* genes contribute to the maintenance of *n*-hexane-tolerance in *E*. *coli*.

### Activities of ROS-scavenging enzymes and susceptibility to H_2_O_2_ and menadione in BW25113∆*katE*∆*katG* and BW25113∆*sodA*∆*sodB*

The levels of catalase and SOD activities in BW25113∆*katE*∆*katG* and BW25113∆*sodA*∆*sodB* were compared with the parent strain BW25113 (Table 4). Most of the catalase and SOD activities were eliminated in BW25113∆*katE*∆*katG* and BW25113∆*sodA*∆*sodB*, respectively. The slight remaining activities of catalase and SOD seemed to be attributable to AhpCF and SodC, respectively. These results showed good agreement with a previous report (Alhama et al. 1998).

**Table 4:** Catalase and SOD activities of *E*. *coli* strains

Strain	Enzymatic activity	
	Catalase (U/mg)	SOD (U/mg)
BW25113	1.41 ± 0.07	0.18 ± 0.01
BW25113∆*katE*∆*katG*	0.06 ± 0.07	0.20 ± 0.03
BW25113∆*sodA*∆*sodB*	1.55 ± 0.15	0.01 ± 0.01

Data represent the mean ± SD of triplicate experiments

Susceptibility of the mutants to ROS was also confirmed by measuring the cell viability after exposure to H_2_O_2_ and a redox-cycling agent, menadione (Additional file 1: Fig. S1). The survival fraction of BW25113∆*katE*∆*katG* in 4 mM H_2_O_2_ and BW25113∆*sodA*∆*sodB* in 60 mM menadione were 40% and 0.3% of those of the parent strain BW25113, respectively.

### Growth of the *E*. *coli* mutants in liquid medium in the presence of organic solvents

**Fig. 3:**
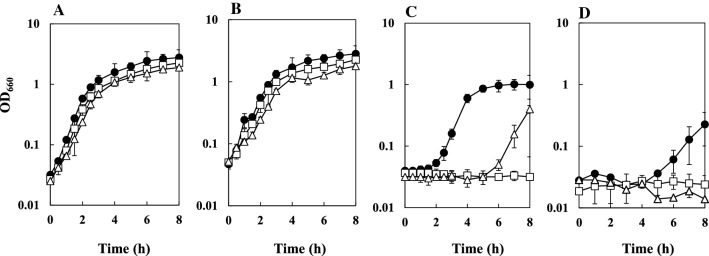
Growth of *E*. *coli* BW25113, BW25113∆*katE*∆*katG* and BW25113∆*sodA*∆*sodB* in LBGMg liquid medium at 37 °C in the absence of an organic solvent (**A**) and in the presence of 10% (vol/vol) cyclooctane (**B**), 10% (vol/vol) *n*-hexane (**C**), or a 10% (vol/vol) *n*-hexane and cyclohexane mixture (9:1 vol/vol) (**D**). **A** 100-μl culture of an overnight-grown *E. coli* strain was inoculated into 10 ml of fresh LBGMg liquid medium containing an organic solvent. This two-phase culture was incubated at 37 °C. Growth was monitored by measuring turbidity (OD_660_). Symbols: filled circle, BW25113; open square, BW25113∆*katE*∆*katG*; open triangle, BW25113∆*sodA*∆*sodB*. Values indicate the means and standard deviations of the results from three independent experiments

The cell growth of BW25113, BW25113∆*katE*∆*katG* and BW25113∆*sodA*∆*sodB* in the LBGMg liquid medium in the presence of a hydrophobic solvent including cyclooctane, *n*-hexane, or a hydrophobic solvent mixture of *n*-hexane and cyclohexane (9:1 vol/vol) was examined by measuring the turbidity (Fig. 3). In the absence of the solvent, the specific growth rates of BW25113, BW25113∆*katE*∆*katG* and BW25113∆*sodA*∆*sodB* during the exponential growth phase were 1.6 h^−1^, 1.5 h^−1^ and 1.3 h^−1^, respectively. In the presence of cyclooctane, the growth rates of BW25113, BW25113∆*katE*∆*katG* and BW25113∆*sodA*∆*sodB* were 1.5 h^−1^, 1.2 h^−1^ and 1.0 h^−1^, respectively. Thus, the growth of these mutants without any organic solvents and in the presence of cyclooctane was slower than that of the parent strain BW25113. The growth of BW25113∆*katE*∆*katG* and BW25113∆*sodA*∆*sodB* was highly suppressed compared to that of BW25113 in the presence of *n*-hexane or the mixture of *n*-hexane and cyclohexane. These results suggested that each of catalase and SOD is involved in the maintenance of tolerance to hydrophobic organic solvents. BW25113∆*sodA*∆*sodB* exhibited an extended lag phase and BW25113∆*katE*∆*katG* did not grow during 8 h cultivation in the presence of *n*-hexane. Thus, BW25113∆*katE*∆*katG* was more sensitive to *n*-hexane than BW25113∆*sodA*∆*sodB*.

### Detection of ROS in *E*. *coli* cells exposed to hydrophobic organic solvents

**Fig. 4:**
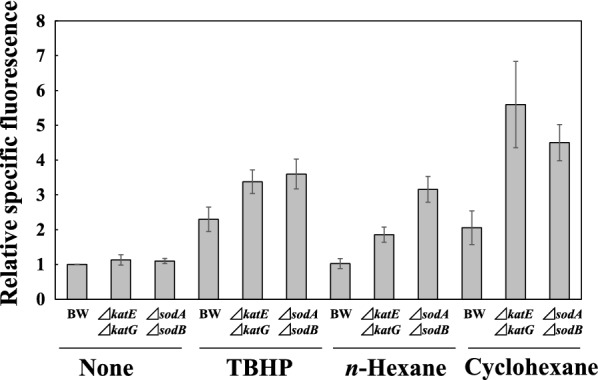
Levels of intracellular ROS in *E*. *coli* cells measured by using carboxy-H_2_DCFDA. ROS levels were measured in the strains exposed to TBHP (*tert*-butyl hydroperoxide), *n*-hexane or cyclohexane as described in the Methods. The relative specific fluorescence shows the ratio of the specific fluorescence in each strain divided by that in strain BW25113 not exposed to the organic solvent. Abbreviations: BW, BW25113; ∆*katE*∆*katG*, BW25113∆*katE*∆*katG*; ∆*sodA*∆*sodB*, BW25113∆*sodA*∆*sodB*. Mean values and standard deviations for three independent experiments are shown

We examined the ROS levels in BW25113, BW25113∆*katE*∆*katG* and BW25113∆*sodA*∆*sodB* in the presence of *n*-hexane or cyclohexane with carboxy-H_2_DCFDA, a fluorescent indicator for ROS in cells (Fig. 4). No significant difference in ROS levels was observed among these three strains in the absence of TBHP (a known inducer for ROS) or organic solvents. Addition of TBHP enhanced the ROS levels in these three strains. Treatment with *n*-hexane had little impact on ROS levels in BW25113 but markedly elevated ROS levels in BW25113∆*katE*∆*katG* and BW25113∆*sodA*∆*sodB*. The ROS levels by exposure to *n*-hexane in BW25113∆*katE*∆*katG* and BW25113∆*sodA*∆*sodB* were 1.9- and 3.2-fold higher than that in BW25113, respectively. Cyclohexane-exposure significantly increased ROS levels in all strains. The ROS levels by exposure to cyclohexane in BW25113∆*katE*∆*katG* and BW25113∆*sodA*∆*sodB* were 2.8- and 2.3-fold higher than that in BW25113, respectively. The ROS levels in the three strains by the addition of cyclohexane were higher than those by addition of *n*-hexane, respectively.

### Induction of *katE*,* katG*, *sodA* and *sodB* by *n*-hexane

**Fig. 5:**
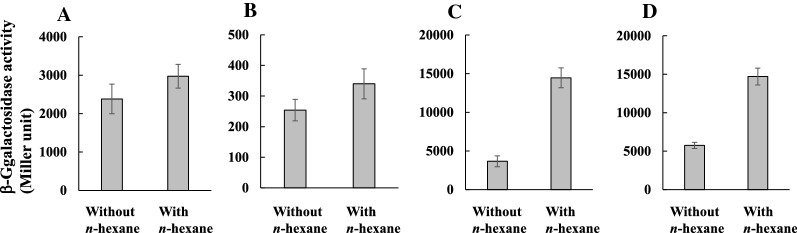
Effect of *n*-hexane on the promoter activity of *katE, katG*, *sodA*, or *sodB*. **A** 100-μl culture of the overnight-grown strain BW25113(pMCkatEp) (**A**), BW25113(pMCkatGp) (**B**), BW25113(pMCsodAp) (**C**), or BW25113(pMCsodBp) (**D**) was inoculated into 10 ml of fresh LBGMg liquid medium containing 50 μg/ml ampicillin without or with 1 ml of *n*-hexane. The culture was incubated at 37 °C until reaching an OD_600_ of approximately 0.6. Cells were treated with chloroform and assayed for β-galactosidase activity. Values indicate the means and standard deviations of the results from three independent experiments

We constructed plasmids pMCkatEp, pMCkatGp, pMCsodAp and pMCsodBp containing *katE*–*lacZ*, *katG*–*lacZ*, *sodA*–*lacZ*, and *sodB*–*lacZ*-fused genes, respectively. BW25113 harboring one of the plasmids was assayed for plasmid-borne β-galactosidase activity (Fig. 5). Promoter activities of *katE*, *katG*, *soda*, and *sodB* with *n*-hexane were 1.2-, 1.3-, 3.9-, and 2.6-fold compared to those without any solvent, respectively. In particular, the promoter activities of *sodA* and *sodB* were significantly increased by exposure to *n*-hexane.

## Discussion

The cell membrane is the main target of organic solvents (Aono et al. 1994). The cytoplasmic membrane of bacterial cells plays a crucial role in various cell functions such as regulation of solutes (nutrients and ions) passage, energy generation, synthesis of membrane lipids and cell wall, secretion of extracytoplasmic proteins, and turgor pressure. Organic solvent molecules intercalate into the lipid bilayer of the membrane. Accumulation of organic solvent in the membrane disturbs a variety of vital cell processes, including those described above. The inner membrane in *E*. *coli* contains an electron transport chain that is the major site of the premature electron leakage to oxygen that generates superoxide (O_2_^−^). Disturbance of the electron transport chain by organic solvent can increase the level of ROS. ROS have the capacity to damage various biomolecules, including proteins, ribosomes and DNA, and to reduce cellular culturability (Imlay 2003).

In the present study, we investigated the involvement of ROS-scavenging enzymes in hydrophobic organic solvent-tolerance. The colony-forming efficiency of *E*. *coli* strains on LBGMg agar showed that the tolerances to *n*-hexane in single gene-knockout mutants lacking the *katE*, *katG*, *ahpF*, *sodA*, *sodB*, or *sodC* gene were similar to that in the parent strain BW25113 (Fig. 1). AhpF is the peroxiredoxin reductase component of alkyl hydroperoxide reductase (AhpCF) that has been reported to be involved in tolerance to organic solvents such as 1,2,3,4-tetrahydronaphthalene (tetralin), cyclohexane, propylbenzene, and 1,2-dihydronaphthalene (Ferrante et al. 1995). AhpF and AhpC proteins act together (Li Calzi and Poole 1997). AhpF utilizes NADH or NADPH as electron donor to AhpC, which converts alkylhydroperoxides to their respective alcohol forms. AhpC is specifically reduced by AhpF and cannot be reduced by other electron transfer systems such as thioredoxin reductase. However, our results showed that deficiency of *ahpF* did not influence the tolerance to *n*-hexane. It has been reported that a *sodA sodB* double mutant was much more sensitive to paraquat than the wild type, although the absence of only the *sodA* gene or only the *sodB* gene had no effect on the sensitivity to paraquat (a superoxide generator) (Carlioz and Touati 1986). Both the *sodA sodB* double mutant and *katE katG* double mutant were more sensitive to *p*-nonylphenol (an endocrine disruptor) than the parent strain (Okai et al. 2004). In particular, the *sodA sodB* double mutant was highly sensitive to *p*-nonylphenol. These findings prompted us to construct BW25113∆*katE*∆*katG* and BW25113∆*sodA*∆*sodB* and then examine their organic solvent-tolerances. BW25113∆*katE*∆*katG* and BW25113∆*sodA*∆*sodB* lost most of their catalase and SOD activities, respectively (Table 4). In addition, we confirmed that BW25113∆*katE*∆*katG* and BW25113∆*sodA*∆*sodB* became sensitive to H_2_O_2_ and menadione (an O_2_^−^ generator), respectively (Additional file 1: Fig. S1). Both BW25113∆*katE*∆*katG* and BW25113∆*sodA*∆*sodB* were highly sensitive to *n*-hexane and a mixture of *n*-hexane and cyclohexane (Figs. 1 and  3). These results showed that accumulation of either H_2_O_2_ or O_2_^−^ in *E*. *coli* can exhibit an inhibitory effect on the cell growth. H_2_O_2_ and O_2_^−^ are relatively weak cytotoxic radical oxygens compared to other radical oxygens such as hydroxyl radicals (Bruno-Barcena et al. 2010; Fridovich 1986). Therefore, the hydroxyl radical produced via Fenton reaction and Haber–Weiss reaction from H_2_O_2_ and O_2_^−^ might be a main cause of the cytotoxicity by addition of hydrophobic organic solvents. Our assays using the fluorescent probe carboxy-H_2_DCFDA showed an increase in ROS after solvent stress (Fig. 4). In addition, the ROS levels in *E*. *coli* cells induced by exposure to cyclohexane were higher than those observed by exposure to *n*-hexane. Thus, the ROS level in *E*. *coli* cells is likely to depend on the amount of organic solvents accumulated in the cells, since a larger amount of cyclohexane than *n*-hexane is accumulated in cells in an organic-aqueous two-liquid-phase system (Tsukagoshi and Aono 2000).

We found that the promoter activities of *sodA* and *sodB* were significantly increased by *n*-hexane (Fig. 5). Expression of SodA is regulated by several global transcription regulators, including the MarA/SoxS/Rob system and Fur (Ferric uptake regulator), and responds to changes in oxygen concentration, redox active compounds, and iron concentration (Fee 1991; Semsey 2014). SodB levels were relatively insensitive to changes in these conditions. SodB seems to be responsible for protection of a cytoplasmic superoxide-sensitive enzyme, while SodA is more effective in preventing DNA damage (Hopkin et al. 1992). Oxidative stress response genes in bacteria are often upregulated during exposure to solvents. The *sodA* gene in *E*. *coli* strains was upregulated by ethanol- or *n*-butanol-induced stress (Cao et al. 2017; Rutherford et al. 2010). Antioxidant enzymes such as catalase and superoxide dismutase in *Pseudomonas putida* showed increased activity upon exposure to toluene (Choi et al. 2014).

In this study, we showed that ROS-scavenging enzymes significantly contributed to the maintenance of tolerance to hydrophobic organic solvents in *E*. *coli*. Various mechanisms of organic solvent-tolerance in *E*. *coli* have been reported so far. These include the multidrug efflux pump (Tsukagoshi and Aono 2000; Watanabe and Doukyu 2012, 2014), maintenance of the proton motive force (Kobayashi et al. 1998), lipopolysaccharides (Abe et al. 2003), fatty acids synthesis (Oh et al. 2012), metabolic pathway for carbon catabolism (Shimizu et al. 2005), reduction of alkylhydroperoxide (Ferrante et al. 1995) and osmoprotectant transport (Doukyu et al. 2012). However, the involvement of catalase and SOD in hydrophobic organic solvent tolerance in *E*. *coli* has not been reported so far. Thus, the present study provides valuable new knowledge of the organic solvent-tolerance mechanisms in *E*. *coli*.

## Supplementary Information

**Additional file 1: Figure S1.** Effects of H_2_O_2_ and menadione on the cell viability of *E. coli* BW25113 and its mutants deficient in ROS-scavenging enzymes. Each strain was exposed to H_2_O_2_ (A) and menadione (B). After incubation with H_2_O_2_ and menadione for 1 h, viable cells were measured by examining the formation of colonies on LB agar medium. The survival fraction was calculated as the number of colonies treated with H_2_O_2_ or menadione divided by that of untreated cells. Symbols: filled circles, BW25113; open squares, BW25113∆*katE*∆*katG*; open triangles, BW25113∆*sodA*∆*sodB*. Values indicate the means and standard deviations of the results from three independent experiments.

## Data Availability

All discussed data have been included into the manuscript or in the Additional file 1. Please turn to the corresponding author for all other requests.

## Abbreviations

ROS: Reactive oxygen species
SOD: Superoxide dismutase
HP: Hydroperoxidase
Carboxy-H_2_DCFDA: 5-(And-6)-carboxy-2’,7’-dichlorodihydrofluorescein diacetate
Km: Kanamycin
EDTA: Ethylenediaminetetraacetic acid
TBHP: *tert*-Butyl hydroperoxide
