# Supplementary material for: Involvement of catalase and superoxide dismutase in hydrophobic organic solvent tolerance of Escherichia coli
Source: AMB Express. 2021 Jun 29;11:97. doi: 10.1186/s13568-021-01258-w (PMC8241964; doi:10.1186/s13568-021-01258-w)
Supplement: Supplementary file 1 — Additional file 1: Figure S1. Effects of H2O2 and menadione on the cell viability of E. coli BW25113 and its mutants deficient in ROS-scavenging enzymes. Each strain was exposed to H2O2 (A) and menadione (B). After incubation with H2O2 and menadione for 1 h, viable cells were measured by examining the formation of colonies on LB agar medium. The survival fraction was calculated as the number of colonies treated with H2O2 or menadione divided by that of untreated cells. Symbols: filled circles, BW25113; open squares, BW25113∆katE∆katG; open triangles, BW25113∆sodA∆sodB. Values indicate the means and standard deviations of the results from three independent experiments. [file 13568_2021_1258_MOESM1_ESM.docx]

**Additional file 1**

**Involvement of catalase and superoxide dismutase in hydrophobic organic solvent tolerance of *Escherichia coli***

Noriyuki Doukyu^1,2*^ and Katsuya Taguchi ^1,2^

Department of Life Science, Toyo University, 1-1-1 Izumino, Itakura-machi, Gunma, 374-0193, Japan^1^

Bio-Nano Electronic Research Center, Toyo University, 2100, Kujirai, Kawagoe, Saitama, 350-8585, Japan^2^

^*^Corresponding author

Noriyuki Doukyu

Department of Life Science, Toyo University, 1-1-1 Izumino, Itakura-machi, Gunma, Japan

E-mail: dokyu@ toyo. jp

Tel.: +81-276-829219

FAX: +81-276-829219


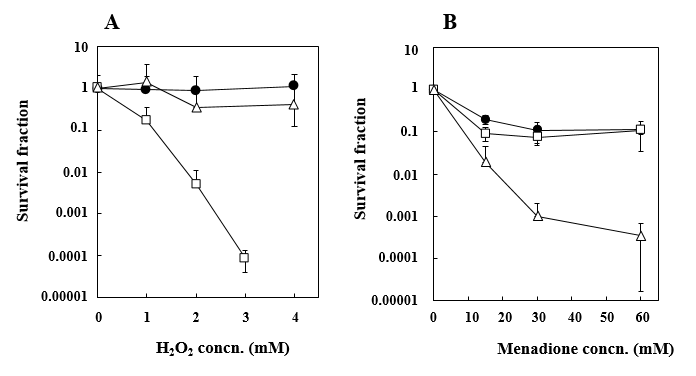


**Figure S1** **Effects of H_2_O_2_ and menadione on the cell viability of *E. coli* BW25113 and its mutants deficient in ROS-scavenging enzymes.** Each strain was exposed to H_2_O_2_ (A) and menadione (B). After incubation with H_2_O_2_ and menadione for 1 h, viable cells were measured by examining the formation of colonies on LB agar medium. The survival fraction was calculated as the number of colonies treated with H_2_O_2_ or menadione divided by that of untreated cells. Symbols: filled circles, BW25113; open squares, BW25113Δ*katE*Δ*katG*; open triangles, BW25113Δ*sodA*Δ*sodB*. Values indicate the means and standard deviations of the results from three independent experiments.
